# The Correlation of a Novel Photographic Parameter for Facial Profile Assessment in Subjects With Different Sagittal Malocclusions: A Prospective Study

**DOI:** 10.7759/cureus.44553

**Published:** 2023-09-01

**Authors:** Rama Raji Sankaranarayanan, Ravindra Kumar Jain

**Affiliations:** 1 Department of Orthodontics and Dentofacial Orthopedics, Saveetha Dental College and Hospitals, Chennai, IND; 2 Department of Dentistry, Saveetha Dental College and Hospitals, Chennai, IND

**Keywords:** profile malocclusions, fsa angle, digital photographs, cephalometric angles, cephalometry

## Abstract

Aim

The soft tissue paradigm shift is the current trend in orthodontic diagnosis and treatment planning. This study’s aim was to assess the correlation of newly derived photographic Frankfort horizontal plane-subnasale to soft tissue pogonion (FSA) angle with other established soft tissue cephalometric angles, such as the Z angle and the Holdaway (H) angle, for estimating facial profile convexity in subjects with all classes of sagittal malocclusions.

Materials and methods

This prospective study included a sample of 60 Dravidian population subjects consisting of 30 males and 30 females with different skeletal sagittal malocclusions (Class I, Class II, and Class III) based on the radiographic criteria (ANB angle). The Z and Holdaway angles on lateral cephalograms were compared with the FSA angles in cephalograms and digital profile photographs. Statistical analysis was done using the Statistical Package for Social Sciences (SPSS) software version 23.0 (IBM SPSS Statistics, Armonk, NY). Pearson’s correlation was done to assess the correlation between soft tissue FSA angle on digital photographs and cephalometric angle (Z angle and Holdaway angle).

Results

The overall Pearson’s correlation was significant (p < 0.05) between the Z and FSA angles in Class I, II, and III malocclusions, which had a high positive correlation. There was a significant positive correlation (p < 0.05) between the Holdaway and FSA angles in subjects with Class I and Class II malocclusions. A moderate positive correlation was noted between the Holdaway and FSA angles in Class III.

Conclusion

Photographic FSA angle can be used to evaluate the facial profile of subjects with different sagittal malocclusions. This angle has a good correlation with other cephalometric profile measures, such as the Z and Holdaway angles used to assess facial profile convexity.

## Introduction

For the past few decades, orthodontic treatment planning has revolved around improving facial attractiveness [1,2]. Cephalometric landmarks and photographic parameters are used in orthodontics for diagnosis [3,4]. After the invention of cephalometry, hard tissue cephalometric norms became the gold standard for orthodontic treatment planning. However, for the past few decades, there has been a shift to the soft tissue paradigm, which emphasizes achieving an esthetic appearance and a balanced profile [5,6]. Soft tissue thickness does not entirely depend on underlying hard tissues; it also depends on age, gender, growth pattern, and underlying malocclusion. Hence, soft tissue assessment is integral to orthodontic treatment planning [7-11].

Soft tissue profiles can be assessed clinically or using cephalograms, digital photographs, and profile silhouettes. Many soft tissue cephalometric norms have been reported in the literature to diagnose and curate treatment keeping in mind the facial esthetics [12-14]. Soft tissue changes after orthodontic treatment have been extensively assessed by many authors [15-17]. Recently, studies have reported assessing soft tissue profiles with photographs and silhouettes [18,19]. Photographs are better than silhouettes, as various soft tissue cephalometric landmarks can be located and overall facial attractiveness can be assessed [20].

The Frankfort horizontal plane-subnasale to soft tissue pogonion (FSA) angle is a soft tissue cephalometric angle newly introduced by Sreenivasagan and Sivakumar and is formed by two planes: Frankfurt horizontal (FH) and subnasale to soft tissue pogonion (SA) [21]. The FSA angle was proposed considering the variations of the N point as a reference point for assessing the soft tissue profile. The study’s aim was to correlate the photographic FSA angle with other established soft tissue angles for assessing the soft tissue profile of subjects with various sagittal skeletal malocclusions.

## Materials and methods

This prospective cross-sectional study was performed on 60 patients of the Dravidian population who reported to the university dental hospital for orthodontic treatment from August 2022 to December 2022. Ethical committee approval was obtained from the Scientific Review Board of Saveetha Institute of Medical and Technical Sciences. The mean age of the included subjects was 26.5 ± 4.5 years, including 30 males and 30 females. Subjects with no history of previous orthodontic treatment or orthognathic surgery were included in the study. Subjects with facial asymmetry or craniofacial deformities of the face were excluded from the study. Informed consent was taken from all the subjects for using their photos and radiographs. Two hundred patients were screened, and 60 subjects were included using simple random sampling. The sample size was calculated using G*Power software version 3.1.9.6 (Universitat Kiel, Kiel, Germany) with alpha error set at 0.05 and power set at 90% based on the study, and each group was equally divided into sagittal skeletal Classes I, II, and III based on the ANB angle [22]. Written informed consent was obtained from all subjects regarding the participation and publication of the data for research and educational purposes. To avoid observer bias, the grouping was done by one observer (RK), and the measurement of the parameters was done by another observer (RR). Overall, favorable intra- and interexaminer reliability for both cephalometric and photographic angles was achieved. The values from two sets of cephalometric analyses were used to calculate the intraexaminer intraclass correlation coefficient (ICC), and the average value of two sets of cephalometric analyses from each examiner was used to calculate the interexaminer ICC. The intra- and interexaminer ICCs of each parameter were calculated. The intra- and interexaminer ICC for x, y, and z coordinates for all landmarks was >0.95 with a confidence interval of 0.88-0.99.

**Figure 1:**
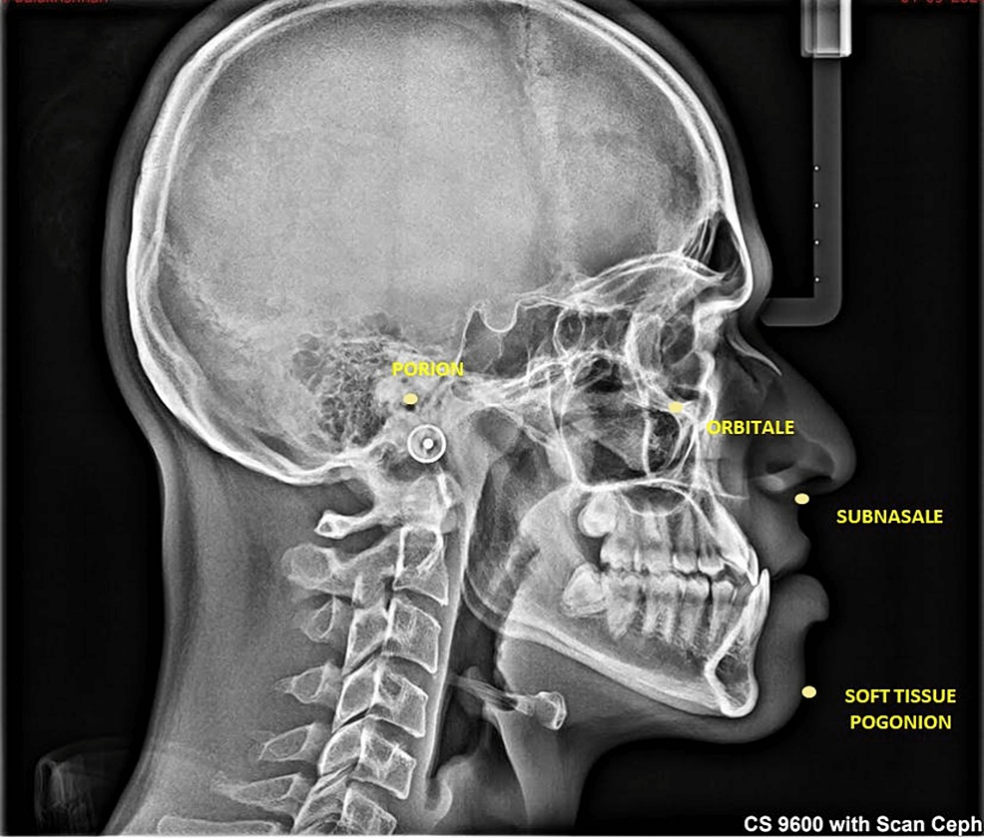
Lateral cephalogram showing soft and hard tissue landmarks.

The lateral cephalograms of all subjects were taken using standard exposure parameters of 83 kV, 10 mA, and 8.0 seconds. Lateral cephalograms were taken in the natural head position (NHP) with the teeth in maximum intercuspation and the lips in repose. Lateral cephalograms were viewed and calibrated in the Dolphin software (Dolphin Imaging and Management Solutions, Chatsworth, CA). The soft tissue cephalometric points were marked (Figure 1), and the Z and Holdaway (H) angles were measured.

**Figure 2:**
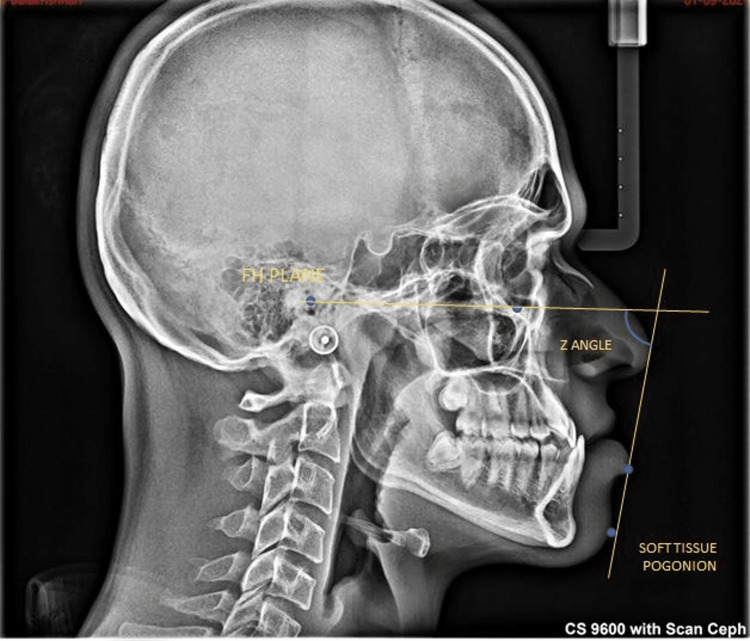
Lateral cephalogram showing the angular parameter, Z angle. It is an angle between the Frankfurt horizontal (FH) plane and a line from the soft tissue pogonion to the most procumbent lip.

The Z angle by Merrifield was formed by connecting the profile line, which is a tangential line from the soft tissue chin to the most anterior point of the most protrusive lip (upper or lower) extending upward to intersect the Frankfort horizontal plane (FHP) (Figure 2) [8].

**Figure 3:**
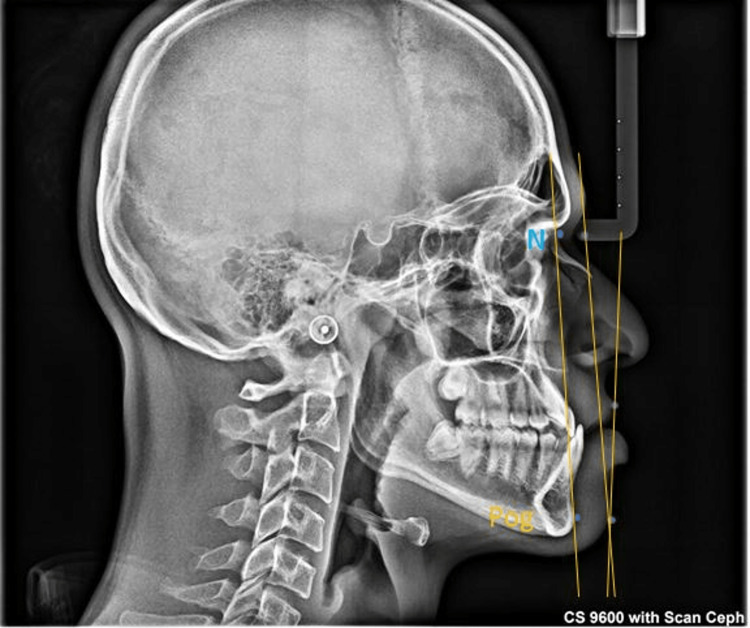
Lateral cephalogram showing the angular parameter, Holdaway (H) angle. The H line is formed by drawing a tangent from the soft tissue pogonion to the upper lip. The H angle is the angle between the H line and N-Pog. N-Pog: nasion and pogonion

The H angle is formed between the soft tissue facial plane to the tangent from the soft tissue pogonion to the most protrusive part of the upper lip (Figure 3) [23].

**Figure 4:**
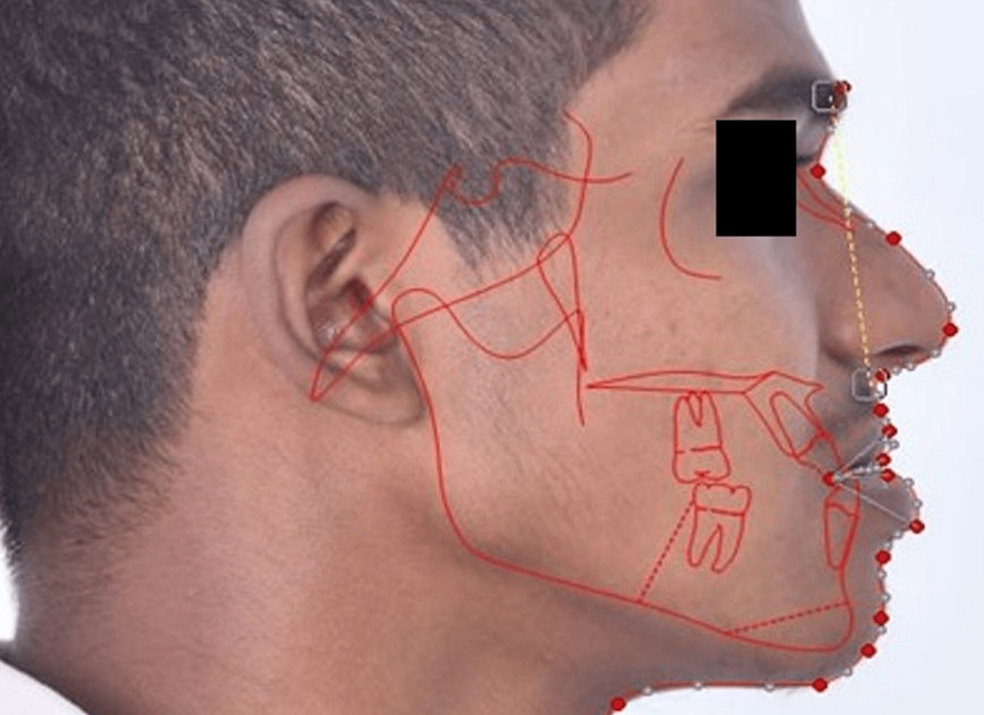
Superimposition of lateral cephalogram over digital photograph in the Dolphin software.

All subjects’ profile photographs were taken using a Nikon SLR D5600 (Nikon Corporation, Tokyo, Japan) digital camera mounted with a macro lens (Nikon EF 100 mm f/2.8 Macro USM). The subjects were made to stand in natural head position (NHP) with the teeth in maximum intercuspation and the lips in repose. To ensure that the subjects were in NHP, the standardization of both photographs and radiographs was done by making the patient stand before a full-size mirror, and they were asked to look at the mirror in a relaxed position to reproduce the NHP in all the subjects. The camera was fixed on a tripod, and profile photographs were taken using standardized settings with 1:1 magnification. The photographs were superimposed over their respective lateral cephalograms. Since the lateral cephalograms were calibrated, this automatically calibrates the profile photographs in the Dolphin software. FH plane was marked on the profile photographs (Figure 4).

**Figure 5:**
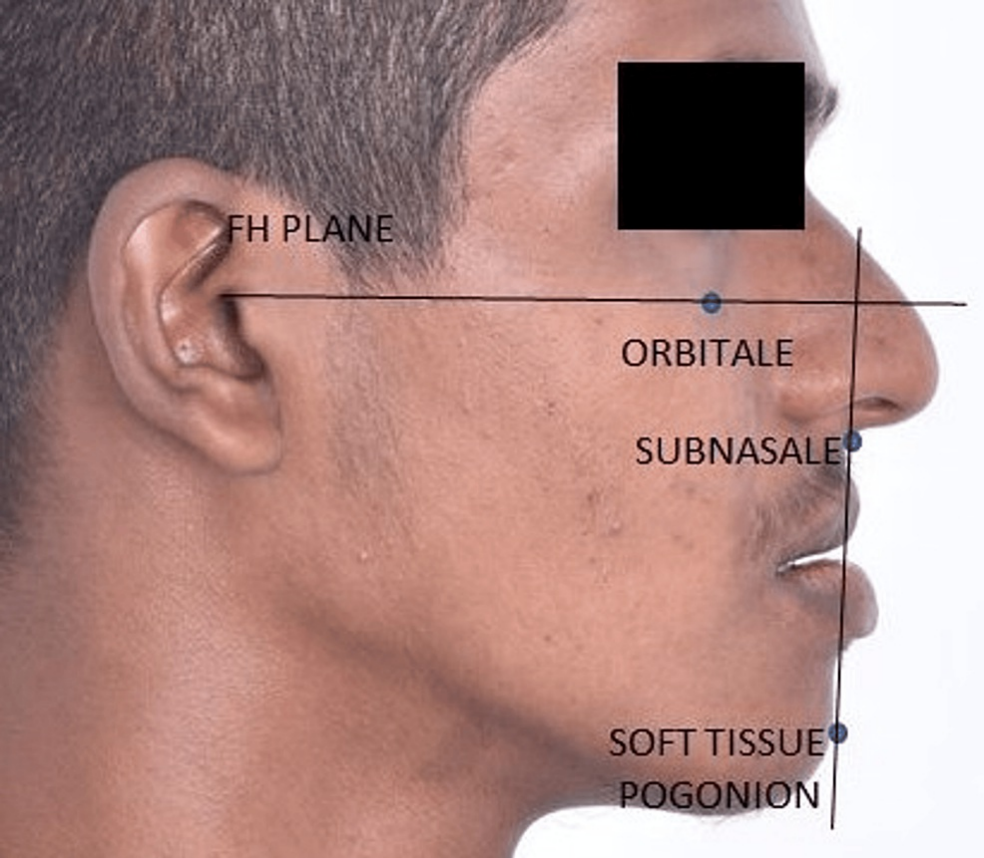
Digital photograph showing the angular parameter, FSA angle. The FSA angle is the angle between the subnasale to soft tissue pogonion (SA) plane and the FHP. FSA, Frankfort horizontal plane-subnasale to soft tissue pogonion; FHP, Frankfort horizontal plane

Both the soft tissue pogonion and subnasale were located on the photographs, and the SA plane was drawn. The photographs’ transparency was restored, and the FSA angle was measured using the annotation tool in the Dolphin software (Figure 5). The FSA angle is the angle between the subnasale to soft tissue pogonion (SA) plane and the FHP.

Statistical analysis was performed in the Statistical Package for Social Sciences (SPSS) software version 23.0 (IBM SPSS Statistics, Armonk, NY). The mean and standard deviation (SD) of the Z and H angles from cephalograms and the FSA angle from the profile photographs of all subjects with Class I, II, and III malocclusions were calculated using the software. Correlation between the photographic FSA, Z, and H angles was done using Pearson’s correlation test.

## Results

**Figure 6:**
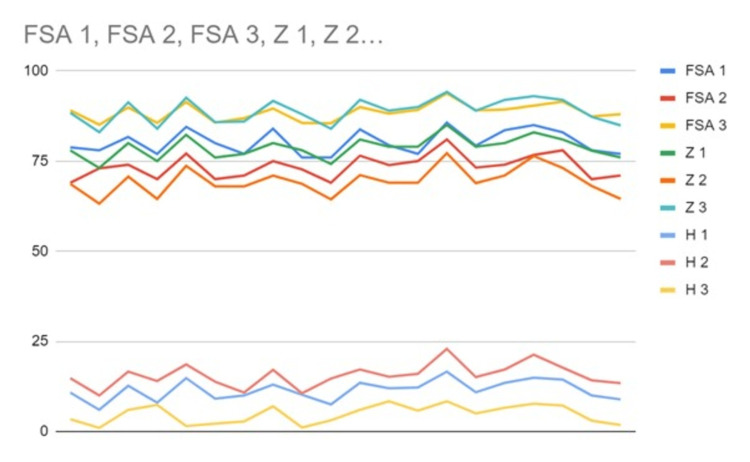
Line chart showing the correlation of FSA angle (Class I, Class II, and Class III) malocclusion with Z angle (Class I, Class II, and Class III) and Holdaway (H) angle (Class I, Class II, and Class III). FSA: Frankfort horizontal plane-subnasale to soft tissue pogonion

The demographic data of all the subjects with Class I, II, and III malocclusions is provided in Table 1, which consists of 60 participants out of which 30 were males and 30 were females. The 60 participants included in the study were divided into Class I, Class II, and Class III groups each consisting of 20 participants. The mean and standard deviation of the FSA, Z, and H angles for Class I, II, and III subjects are given in Table 2. Table 3 and the scatter plot diagram show that Pearson’s correlation was used to assess the correlation quotient between the FSA, Z, and H angles (Figure 6). Pearson’s correlation between the Z angle and FSA angle is strongly correlated in Class I, II, and II malocclusions with r-values of 0.8, 0.8, and 0.9, respectively. However, the Holdaway angle is strongly correlated with the FSA angle in Class I and Class II malocclusions with r-values of 0.8 and 0.7, respectively. The Holdaway angle is moderately correlated with the FSA angle in the Class III malocclusion group with an r-value of 0.5.

**Table 1:** Demographic data.

	Male	Female
Class I	9 (45%)	11 (55%)
Class II	8 (40%)	12 (60%)
Class III	13 (65%)	7 (35%)

**Table 2:** Mean and standard deviation of the FSA angle, Z angle, and H angle in all three groups (Class I, Class II and Class III). FSA, Frankfort horizontal plane-subnasale to soft tissue pogonion; SD, standard deviation; H, Holdaway

Malocclusion type	FSA angle mean and SD	Z angle mean and SD	H angle mean and SD
Class I	80.24 ± 3.32	78.72 ± 3.00	11.45 ± 2.78
Class II	73.50 ± 3.28	69.46 ± 3.77	15.56 ± 3.25
Class III	88.57 ± 2.35	88.91 ± 3.42	4.60 ± 2.42

**Table 3:** Pearson’s correlation matrix showing bivariate correlation among the FSA angle, Z angle, and H angle (p value < 0.05). FSA, Frankfort horizontal plane-subnasale to soft tissue pogonion; H, Holdaway

Groups		FSA and Z angle (r-value)	FSA and H angle (r-value)
Class I	Pearson’s correlation	0.836	0.85
	Significance	0.001	0.002
Class II	Pearson’s correlation	0.821	0.743
	Significance	0.003	0.001
Class III	Pearson’s correlation	0.904	0.553
	Significance	0.002	0.012

## Discussion

There has been a soft tissue paradigm shift in orthodontic diagnosis and treatment planning with more emphasis on soft tissue esthetics [24]. Soft tissue profile assessment plays a crucial role in orthodontic diagnosis and treatment planning [25]. Soft tissue profile analysis can be assessed using clinical evaluation, radiographs, photographs, and silhouettes [26]. The FSA angle is a soft tissue angle with the Frankfort horizontal plane as a reference plane. In this study, the FSA angle was correlated with other already established soft tissue profile cephalometric angles, and a significant correlation between FSA and other cephalometric soft tissue angles was noted in all malocclusions.

The present study included a sample of 60 subjects with a mean age of 26.5 ± 4.5. Subjects with different skeletal malocclusions (Classes I, II, and III) were included to assess the correlation of the FSA angle with other routinely used soft tissue angles. The Z and Holdaway angles were chosen for assessing the soft tissue maxillomandibular discrepancy. Both these angles were chosen because they are widely used for soft tissue profile analysis [27]. A strong correlation was noted between the FSA angle and Z angle in Class I (r-value = 0.836), Class II (r-value = 0.821), and Class III malocclusion subjects (r-value = 0.904; Table 3). A previous study by Derwich et al. reported that the Z angle could be used as an accurate diagnostic parameter to assess the convexity of the facial profile [27]. Contini et al. reported that the Z angle could reliably assess facial convexity in different malocclusions with various growth patterns [28]. In the present study, the FSA angle coincides with both the soft tissue cephalometric angle in most of the malocclusions; hence, the FSA angle can be used to assess facial profile convexity (Table 3 and Figure 3).

In this study, it was noted that the FSA angle strongly correlated with the Holdaway angle in Class I and II malocclusions (r-value = 0.85 and r-value = 0.74); however, there was a moderate correlation in Class III malocclusions (r-value = 0.553). The soft tissue compensations for the hard tissue discrepancy can explain the moderate correlation of the FSA angle with Class III malocclusions. The difference in lip thickness can be explained by results obtained from a study by Yan et al., who correlated upper lip thickness with various skeletal patterns (Classes I, II, and III) and found that the thickness of the upper lip increased in Class III where the soft tissue augments for maxillary retrognathism. In contrast, in Class II malocclusions, the upper lip thickness was in the normal range [29]. The Holdaway angle uses the upper lip to estimate the sagittal discrepancy, unlike the Z angle, which uses the most protrusive lip, thereby underestimating the skeletal discrepancy. This explains the moderate correlation of the FSA angle with the H angle in Class III subjects. Jacobson stated that the N point does not fall on the anterior cranial base but on the sutural junction of the nasal and frontal bone. The variability of the N point is found there both horizontally and vertically, which can lead to variations in soft tissue N point [30]. The FSA angle has the advantage of not involving a reference plane with the N point but instead uses FHP as a reference plane closest to the true horizontal plane [31,32]. The null hypothesis is partially rejected as significant results are established for some of the parameters under study. The FSA and Z angles estimate the soft tissue profile in different malocclusions, whereas the Holdaway angle estimates the soft tissue profile in Classes I and II. However, borderline Class III cases are questionable due to soft tissue thickness. In 7-12-year-old children, the X-ray beam frequently covers an area that is much larger than the area of interest, inadvertently exposing other structures of the maxillofacial, cranial, and neck regions. Structures that are particularly sensitive to radiation such as the brain, eyes, and thyroid gland have been exposed incorrectly due to inappropriate adult program setting on child patients, which includes not using the segmental capability that most dental panoramic machines possess, staff knowledge with regard to correct thyroid shield use, and limited possibilities to collimate the beam in lateral cephalograms. Hence, this particular photographic angle will play a major role in orthodontic diagnosis.

Limitations

The study had a few limitations: first, this study involves only a single ethnicity sampling with a small sample size; second, gender-based conclusions were not derived from the study; and third, the patient grouping was based on the type of sagittal malocclusion not on their growth pattern.

## Conclusions

The photographic FSA angle is a simple non-radiographic alternative in evaluating the facial profile of subjects with different sagittal malocclusions, and it has a good correlation with other cephalometric measures of assessing facial profile convexity. Hence, this soft tissue angle can be used if there is N point variation and to educate the patients to seek orthodontic treatment by quantification with this angle on digital photographs. Though this study is conducted in a single ethnic (Dravidian) population, this could be used in this particular population at the current stage. The FSA angle is a non-radiographic parameter and can be used as an adjunctive chairside tool, which can used for assessing clinical orthodontic profiles using digital profile photographs in day-to-day practice.
